# Pattern of Regional Cortical Thinning Associated with Cognitive Deterioration in Parkinson’s Disease

**DOI:** 10.1371/journal.pone.0054980

**Published:** 2013-01-24

**Authors:** Javier Pagonabarraga, Idoia Corcuera-Solano, Yolanda Vives-Gilabert, Gisela Llebaria, Carmen García-Sánchez, Berta Pascual-Sedano, Manuel Delfino, Jaime Kulisevsky, Beatriz Gómez-Ansón

**Affiliations:** 1 Movement Disorders Unit, Neurology Department, Sant Pau Hospital, Barcelona, Spain; 2 Neuroradiology Unit, Sant Pau Hospital, Barcelona, Spain; 3 Port d’Informació Científica (PIC), Barcelona, Spain; 4 Institut de Física d’Altes Energies (IFAE), Barcelona, Spain; 5 Universitat Autònoma de Barcelona (U.A.B.), Barcelona, Spain; 6 Institut d’Investigacions Biomèdiques – Sant Pau (IIB-Sant Pau), Barcelona, Spain; 7 Centro de Investigación Biomédica en Red sobre Enfermedades Neurodegenerativas (CIBERNED), Barcelona, Spain; The Mental Health Research Institute of Victoria, The University of Melbourne, Australia

## Abstract

**Background:**

Dementia is a frequent and devastating complication in Parkinson’s disease (PD). There is an intensive search for biomarkers that may predict the progression from normal cognition (PD-NC) to dementia (PDD) in PD. Mild cognitive impairment in PD (PD-MCI) seems to represent a transitional state between PD-NC and PDD. Few studies have explored the structural changes that differentiate PD-NC from PD-MCI and PDD patients.

**Objectives and Methods:**

We aimed to analyze changes in cortical thickness on 3.0T Magnetic Resonance Imaging (MRI) across stages of cognitive decline in a prospective sample of PD-NC (n = 26), PD-MCI (n = 26) and PDD (n = 20) patients, compared to a group of healthy subjects (HC) (n = 18). Cortical thickness measurements were made using the automatic software Freesurfer.

**Results:**

In a sample of 72 PD patients, a pattern of linear and progressive cortical thinning was observed between cognitive groups in cortical areas functionally specialized in declarative memory (entorhinal cortex, anterior temporal pole), semantic knowledge (parahippocampus, fusiform gyrus), and visuoperceptive integration (banks of the superior temporal sulcus, lingual gyrus, cuneus and precuneus). Positive correlation was observed between confrontation naming and thinning in the fusiform gyrus, parahippocampal gyrus and anterior temporal pole; clock copy with thinning of the precuneus, parahippocampal and lingual gyrus; and delayed memory with thinning of the bilateral anteromedial temporal cortex.

**Conclusions:**

The pattern of regional decreased cortical thickness that relates to cognitive deterioration is present in PD-MCI patients, involving areas that play a central role in the storage of prior experiences, integration of external perceptions, and semantic processing.

## Introduction

Cognitive decline and dementia have been widely described in Parkinson’s disease (PD). Subtle cognitive defects satisfying criteria for mild cognitive impairment (PD-MCI) are present in up to 25% of PD patients from the early to mid stages of the disease [1], [2]. As the disease evolves, the cumulative prevalence of dementia (PDD) is progressively increasing to 45% ten years after disease onset, and to almost 80% after 20 years [3], [4]. The rate of progression to dementia, however, is not homogeneous. Depending on the age of PD onset, the presence of hallucinations, REM behavior disorder, PIGD motor phenotype, specific genotypes [5], [6], or the development of posterior cortical cognitive defects (e.g., semantic fluency, figure copying) [6], [7], some patients progress more rapidly to dementia [3], [6]. In patients developing dementia within the first 10 years of the disease, PDD has been associated with shorter overall survival and more severe cortical α-synuclein pathology in limbic and neocortical areas [3], [8], [9].

Neuroimaging studies further support the importance of structural changes in limbic and cortical areas in the progression of cognitive impairment in PD. Grey matter loss in the limbic/paralimbic system (amygdala, anterior cingulate cortex, hippocampus and parahippocampal cortex) clearly characterizes dementia in PD [10], [11], and some studies have also found grey matter loss in occipito-parietal areas in PDD [12], [13]. Using voxel-based morphometry (VBM), prior studies analyzing the structural changes present in earlier stages of cognitive deterioration have begun to disclose a pattern of cortical involvement in the temporal, parietal and occipital cortex in PD-MCI patients [14], [15], [16], [17]. Further knowledge of the structural changes of PD-MCI patients would aid in the early detection of patients at risk of developing dementia. Recent advances in image processing and acquisition has allowed the automatic extraction of cortical thickness from MRI [18]. In non-demented PD patients, cortical thickness seems more sensitive than VBM to identify regional grey matter changes [19] and, compared to VBM, provides the most plausible measure of change over time [20].

In order to delineate the structural changes that characterize the progressive nature of cognitive deterioration in PD, we analyzed changes in cortical thickness in healthy controls compared with PD patients with normal cognition (PD-NC), PD-MCI and PDD.

## Materials and Methods

### 2.1 Ethics Statement

This study was approved by the Research Ethics Committee of the Hospital de la Santa Creu i Sant Pau. The ethical committee approved all consent procedures. Written informed consent was obtained from all participants or the patient’s caregiver. A doctor or second party was present to assess each patient’s capacity to consent.

### 2.2 Study Population

Ninety-eight subjects participated in this study. Seventy-seven patients fulfilling research diagnostic criteria for PD [21] (age 72.5±7 years, education 8.5±5 years, disease duration 7.3±5 years) were prospectively recruited from a sample of outpatients regularly attending the Movement Disorders Clinic at Sant Pau Hospital, Barcelona. According to global cognitive status, patients were classified as patients with normal cognition (PD-NC; n = 26), PD-MCI (n = 28), or PDD (n = 23). Cognitive status was assessed by the Clinical Dementia Rating Scale (CDR) [22] and the Mattis Dementia Rating Scale (MDRS) [23]. The MDRS is divided into five subscales measuring attention, initiation and perseveration, construction, conceptualization, and memory. Total score and subscores for each subscale were obtained. According to the diagnostic criteria for PDD proposed by the MDS [4], and published MDRS normative data [24], we classified PD patients as demented when total CDR score was ≥1 and impairment was present in at least two subscores of the MDRS. The diagnosis of PD-MCI was established when total CDR score = 0.5 and only one MDRS subscore was impaired. PD-NC patients had total CDR score = 0 and all MDRS subscores were within the normal range. The score on the CDR was derived from a semistructured interview with the participant and a knowledgeable informant, following the version and scoring rules indicated by Morris et al. [25] The interrater reliability [26] and validity [27] of the CDR have been established by correlation with neuropathologic features observed at autopsy.

The CDR was administered by an experienced neurologist in cognitive defects in Parkinson’s disease (JP), and two neuropsychologists (GLl and CG), who were blinded to the CDR classification, administered the Mattis DRS. PDD patients with severe dementia (CDR = 3) who were unable to follow all the clinical and radiological procedures of the study were excluded. In order to further characterize the neuropsychological characteristics of patients, and to correlate cortical thickness changes with neuropsychological performance, we administered the PD-CRS, a PD-specific neuropsychological battery that enables discrimination of frontal-subcortical from posterior cortical defects [7]. Total, frontal-subcortical, and posterior cortical scores, and scores from every item in the scale were recorded.

Twenty-one healthy subjects, most of whom were spouses or caregivers of the patients, served as the control group (HC). Bonferroni post-hoc comparisons showed that cognitive subgroups (HC versus PD-NC, PD-NC versus PD-MCI, and PD-MCI versus PDD) were well-matched in terms of age and education. As expected, PDD patients were significantly older than both PD-NC (p = 0.03) and HC (p = 0.001), and were less educated than HC (p = 0.006). The PD-NC and PD-MCI groups were also matched for disease duration, motor severity, and dopaminergic doses, but the PDD group had longer disease duration and more severe motor dysfunction. The demographic and clinical characteristics of the sample are described in **Table 1**.

**Table 1:** Demographic and clinical features of the sample.

	PD-NC (n = 26)	PD-MCI (n = 26)	PDD (n = 20)	HC (n = 18)	p (Bonferroni post-hoc comparisons)
Age, y	71.5±4	73.3±7	76.9±4	68.2±4	a 0.31; b 0.66; c 0.14
Sex, %men	55%	59%	52%	53%	0.7 (χ^2^)
Education, y	9.0±5	9.2±4	7.1±4	10.4±4	a 0.42; b 0.99; c 0.11
MDRS total	133.8±7	128±8	110±17	137±4	a 0.74; b 0.03; c 0.001
PD-CRS total	81.6±16	72.9±14	47.9±16	87.7±6	a 0.13; b 0.02; c 0.001
PD-CRS frontal-subcortical	54.4±15	45.6±13	25.1±13	59.2±5	a 0.92; b 0.03; c 0.001
PD-CRS posterior cortical	27.1±2	27.2±2	22.2±2	28.5±1	a 0.85; b 0.99; c 0.001
Disease duration, y	7.3±4	6.8±4	9.6±6	–	b 0.8; c 0.03
UPDRS-III (on)	24±8	21±9	29±10	–	b 0.7; c 0.02
Hoehn &Yahr (on)	2.2±0.4	2.0±0.6	2.8±0.7	–	b 0.7; c 0.08
Total-LEDD, mg/day	791±318	700±399	860±386	–	b 0.3; c 0.1

a HC vs. PD-NC;

b PD-NC vs PD-MCI;

c PD-MCI vs. PDD. PD-NC: Parkinson’s disease with normal cognition; PD-MCI: PD with mild cognitive impairment; PDD: PD with dementia; MDRS: Mattis Dementia Rating Scale; PD-CRS: Parkinson’s Disease-Cognitive Rating Scale; UPDRS: Unified Parkinson’s Disease Rating Scale; LEDD: levodopa equivalent daily dose.

Each patient was interviewed regarding years of formal education, disease onset and medication history, current medications and dosage [LD daily dose, and dopaminergic agonists - LD equivalent daily dose (DA-LEDD)] [28]. Motor status and stage of illness were assessed by the UPDRS-III and Hoehn & Yahr scales [29].

Exclusion criteria included history of major psychiatric disorders, cerebrovascular disease, other conditions known to impair mental status other than PD, or the presence of MRI contraindications (e.g., severe claustrophobia, pacemaker, non-compatible prosthesis). Patients with focal abnormalities on MRI, or non-compensated systemic diseases were also excluded. Eight subjects (3 HC, 2 PD-MCI, 3 PDD) were excluded because of motion artifacts.

In patients with motor fluctuations, cognition was examined during the ‘on’ state. All participants were at stable doses of dopaminergic drugs during the 4 weeks before inclusion.

### 2.3 Data Acquisition

MRI was obtained in all subjects using a 3.0 Tesla Philips Achieva facility, and a dedicated protocol including 3D-MPRAGE of the whole brain (TR = 6.7, TE = 3.1, matrix 288×288×170, voxel size 0.889×0.889×1.2 mm). MR images were deidentified and saved in a dedicated repository, using the PICNIC web tool.

### 2.4 MRI Processing and Analysis

#### Surface reconstructions and estimation of cortical thickness

MRI data were analyzed and the surfaces reconstructed using Freesurfer v4.3.1 (http://surfer.nmr.mgh.harvard.edu), as published [30]. All subjects were affine registered to the Talairach atlas [31], image intensity variations due to magnetic field inhomogeneities were normalized, and a skull stripping algorithm was applied [32].

Then, an estimate of the gray/white boundary was constructed by classifying all white matter voxels in an MRI volume. The surface of the white matter voxels was refined to obtain better accuracy in the gray/white boundary and then subsequently deformed outward to find the pial surface, according to Fischl and Dale [33]. The surface deformation was based on a local adaptive estimation of the MRI values at the different surfaces by minimizing a constrained energy function. Cortical thickness estimates were then obtained with the shortest distance between the white matter and the pial surfaces at each location in the brain.

Subsequently, a masked investigator conducted a visual inspection and, if necessary, the investigator performed a manual correction in order to refine the segmentation and to correct software delineation errors. All of the automated image processing and the visual inspections were performed by a single masked investigator (IC).

### 2.5 Statistical Analysis

Clinical variables were also analysed using SPSS 17.0. One-way ANOVA with post-hoc Bonferroni tests were used to test for between-group differences in demographical, clinical, and neuropsychological variables. In these analyses, statistical threshold was set at p<0.05.

#### Brain structural analysis

Statistical maps were generated using Freesurfer’s QDEC 1.4 (Query, Design, Estimate, Contrast) application. QDEC fits a general linear model (GLM) at each surface vertex to explain the data from all subjects in the study. The results were obtained with a FWHM (full-width/half max) of 10 mm. Significant differences were considered for clusters with a p-value <0.01, uncorrected. The discrimination threshold was reduced at uncorrected p<0.001 to verify areas that distinguished cognitive groups. The results were represented in inflated surfaces in order to allow for improved visualization of sulcal regions while maintaining topology. Freesurfer has the capability to parcellate the cerebral cortex based on arbitrary maps defined on a standard template. For this analysis, we used the Desikan-Killiany cortical atlas, that parcellates the cerebral cortex into 33 cortical regions per hemisphere.

#### Correlation between average cortical thickness areas and neuropsychological variables

FreeSurfer has the ability to compute statistics averaged over a predefined region of interest (ROI). From the statistical maps obtained comparing all participant groups, labels (regions of interest) corresponding to clusters with p<0.05 were drawn. Labels were then mapped to individual subjects of the study. Finally, statistics on each individual label for each subject were created, allowing for computation of the average cortical thickness and standard deviation for the region of interest.

To examine the association between cortical thickness and neuropsychological performance, raw PD-CRS scores were analyzed using bivariate correlations controlling for age, educational level, and disease severity.

## Results

### Cortical Thickness Changes between Cognitive Groups (Table 2, Figure 1)

**Figure 1:**
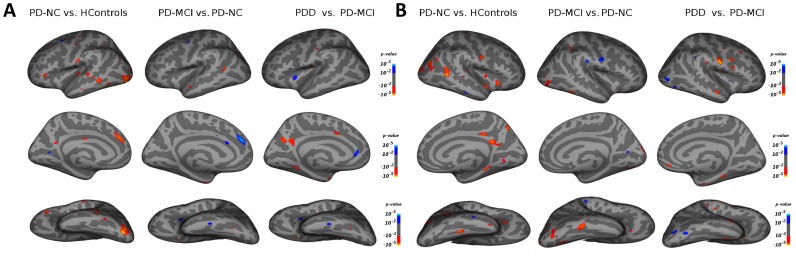
Cortical areas with significant cortical thickness changes between cognitive groups (p<0.01). Red areas show areas of decreased cortical thickness, and blue areas regions with increased cortical thickness. 1A: Left hemisphere; 1B: Right Hemisphere.

**Table 2:** Anatomical location of areas of decreased and increased cortical thickness among controls and Parkinson’s disease patients according to cognitive status.

Talairach Coordinates				Anatomical location	Cluster size,mm^2^	Num. of Vertices	p
x	y	z	side				
PD-NC < HC							
−4	35	25	L	Superior frontal gyrus	298	314	0.001
−65	−43	−3	L	Middle temporal gyrus	246	238	0.001
60	−58	7	R	Middle temporal gyrus	496	589	0.01
1	−46	22	R	Isthmus cingulate	227	359	0.001
42	−78	23	R	Inferior parietal lobule	423	409	0.001
−35	−87	−11	L	Lateral occipital cortex	896	893	0.001
PD-MCI < PD-NC							
−31	0	−37	L	Anterior temporal pole/Entorhinal cortex/Anterior Fusiform gyrus	403	362	0.01
−53	−4	−15	L	Superior temporal gyrus	52	75	0.01
−48	−53	11	L	Banks of the superior temporal sulcus	64	106	0.01
26	−70	−4	R	Fusiform gyrus	103	147	0.01
50	−48	−14	R	Inferior temporal gyrus	181	206	0.001
9	−86	19	R	Cuneus	428	424	0.01
40	−84	−9	R	Lateral occipital cortex	250	217	0.01
PD-MCI>PD-NC							
−5	40	20	L	Superior frontal gyrus	282	285	0.001
−2	23	19	L	Caudal anterior cingulate cortex	27	57	0.01
11	−7	65	R	Precentral	58	94	0.01
PDD < PD-MCI							
6	39	−14	R	Medial orbitofrontal cortex	39	64	0.01
−5	12	33	L	Caudal anterior cingulate cortex	53	98	0.01
−21	3	−27	L	Anterior temporal pole/Entorhinal cortex	131	114	0.01
25	−7	−31	R	Entorhinal cortex	51	74	0.01
33	−33	−12	R	Parahippocampal	48	128	0.01
−32	−13	−28	L	Fusiform gyrus	22	39	0.01
38	−17	−26	R	Fusiform gyrus	98	118	0.01
−29	−46	−3	L	Lingual gyrus	47	148	0.01
−5	−54	26	L	Precuneus	181	303	0.01
PDD >PD-MCI							
−2	40	2	L	Rostral anterior cingulate cortex	102	119	0.01
−35	10	1	L	Insula	98	169	0.01

PD: Parkinson’s disease; PD-NC: PD with normal cognition; PD-MCI: PD with mild cognitive impairment; PDD: PD with dementia; HC: healthy controls; R: right; L: left. Num. of vertices: Number of vertices. The coordinates x, y and z refer to the anatomical location, indicating standard stereotactic space as defined by Talairach and Tournoux. In this table, the reported voxels are p uncorrected <0.01 or <0.001 as indicated.

#### HC versus PD-NC

Although no differences were observed on neuropsychology between HC and PD-NC patients, decreased cortical thickness (p<0.01, GLM corrected for age and education) was observed in the left superior frontal gyrus, left lateral occipital cortex, bilateral middle temporal gyrus, right isthmus of the cingulate cortex, and right inferior parietal lobule.

#### PD-MCI versus PD-NC

Compared with the PD-NC group, PD-MCI patients showed decreased cortical thickness amongst patients (p<0.01, GLM corrected for age and education) in the left anterior temporal pole, entorhinal cortex, fusiform gyrus, superior temporal gyrus, and banks of the superior temporal sulcus (bSTS). On the right side, decreased cortical thickness was also observed in the cuneus, lateral occipital cortex, inferior temporal cortex, and the fusiform gyrus. In addition, the following 3 regions were found to have increased cortical thickness in PD-MCI patients: left superior frontal gyrus, left caudal anterior cingulate cortex, and right precentral region.

#### PDD versus PD-MCI

When compared with PD-MCI, PDD patients showed decreased cortical thickness (p<0.01, GLM corrected for age and education) in the left caudal anterior cingulate cortex, entorhinal cortex, anterior temporal pole, fusiform gyrus, lingual gyrus, and precuneus. On the right side, cortical thinning was found in the medio-orbital frontal cortex, entorhinal cortex, parahippocampus, and fusiform gyrus. Areas with increased cortical thickness were also found in PDD patients, with cortical enlargement in left insula and left rostral anterior cingulate cortex.

Finally, when PDD and PD-NC groups were compared, cortical thinning was observed extensively and bilaterally in both anterior temporal regions (entorhinal, temporal pole, ACC) and medial posterior regions (cuneus, precuneus, lingual gyrus, isthmus of the cingulate cortex).

### Neuropsychological Correlations (Table 3)

**Table 3:** Bivariate correlations between neuropsychological performance and cortical thickness changes.

PD-MCI	Decreased cortical thickness:- Sustained attention - Left bSTS (r = 0.32; p = 0.006)Right cuneus (r = 0.26; p = 0.01)Right fusiform gyrus (r = 0.21; p = 0.001)- Immediate free-recall - Right cuneus (r = 0.23; p = 0.01)verbal memory- Confrontation naming - Right fusiform gyrus (r = 0.24; p = 0.01)
PDD	Decreased cortical thickness:- Sustained attention - Right cuneus (r = 0.36; p = 0.001)Left lingual gyrus (r = 0.33; p = 0.002)Right parahippocampal gyrus (r = 0.34; p = 0.003)- Alternating verbal - Right parahippocampal gyrus (r = 0.38; p = 0.001) fluency Left lingual gyrus (r = 0.32; p = 0.006)Left precuneus (r = 0.23; p = 0.01)- Working memory - Left lingual gyrus (r = 0.31; p = 0.005)Left precuneus (r = 0.26; p = 0.01)Right parahippocampal gyrus (r = 0.28; p = 0.01)Right anteromedial temporal cortex (r = 0.22; p = 0.01)- Delayed free-recall - Right parahippocampal gyrus (r = 0.41; p = <0.001) verbal memory Left anteromedial temporal cortex (r = 0.32; p = 0.005)Right anteromedial temporal cortex (r = 0.26; p = 0.01)Left lingual gyrus (r = 0.28; p = 0.01)- Confrontation naming - Left fusiform gyrus (r = 0.30; p = 0.009)Left temporal pole (r = 0.23; p = 0.01)Right parahippocampal gyrus (r = 0.27; p = 0.01)- Clock copying - Right parahippocampal gyrus (r = 0.41; p<0.001)Left precuneus (r = 0.27; p = 0.01)Left lingual gyrus (r = 0.30; p = 0.01)Increased cortical thickness:- Sustained attention - Caudal anterior cingulate cortex (r = −0.18, p = 0.03)

Bivariate correlations (Pearson’s correlation coefficient; p≤0.01) were used to examine the relationship between performance in PD-CRS neuropsychological items and mean cortical thickness in those areas that significantly differentiated cognitive groups. All the variables explored with Pearson correlations were normally distributed.

Correlations between the entire cortical surface and the PD-CRS within cognitive groups showed that mean left hemisphere cortical thicknes correlated with delayed verbal memory (r = −0.62; p = 0.027), and clock drawing (r = −0.52; p = 0.03) and copying (r = −0.43; p = 0.041) in the PDD group, and with working memory (r = −0.37; p = 0.03), and clock drawing (r = −0.33; p = 0.03) and copying (r = −0.30; p = 0.04) in the PD-MCI group. No correlations were observed between the mean right hemisphere cortical thickness and any clinical score.

In PD-MCI patients, sustained attention correlated with decreased cortical thickness in the left bSTS (r = 0.32; p = 0.006), right cuneus (r = 0.26; p = 0.01), and right fusiform gyrus (r = 0.21; p = 0.01). Immediate free-recall verbal memory correlated with decreased cortical thickness in the right cuneus (r = 0.23; p = 0.01), and confrontation naming with decreased cortical thickness in the right fusiform gyrus (r = 0.24; 0.01).

In PDD patients, a wider pattern of correlations was observed. Positive correlations were found between: (i) sustained attention with the right cuneus (r = 0.36; p = 0.001), with 0.003); (ii) working memory with the left lingual gyrus (r = 0.31; p = 0.005), left precuneus (r = 0.26; p = 0.01), right parahippocampal gyrus (r = 0.28; p = 0.01), and right anteromedial temporal cortex (r = 0.22; p = 0.01); and (iii) alternating verbal fluency with the right parahippocampal gyrus (r = 0.38; p = 0.001), left lingual gyrus (r = 0.32; p = 0.006), and left precuneus (r = 0.23; p = 0.01). When analyzing non-executive tasks, positive correlations were found between: (i) delayed free-recall verbal memory with the right parahippocampal gyrus (r = 0.41; p = <0.001), left (r = 0.32; p = 0.005) and right (r = 0.26; p = 0.01) anteromedial temporal cortex, and left lingual gyrus (r = 0.28; p = 0.01); (ii) confrontation naming with the left fusiform gyrus (r = 0.30; p = 0.009), left temporal pole (r = 0.23; p = 0.01), and right parahippocampal gyrus (r = 0.27; p = 0.01); and (iii) clock copy with the right parahippocampal gyrus (r = 0.41; p<0.001), left precuneus (r = 0.27; p = 0.01), and left lingual gyrus (r = 0.30; p = 0.01).

Finally, a weaker correlation was observed between increased cortical thickness in the caudal anterior cingulate cortex and lower scores in sustained attention (r = −0.18, p = 0.03). No further correlations with neuropsychological items were observed for changes in cortical thickness in the cingulate cortex or prefrontal areas, and no correlation was found between cortical thickness changes in the PD-NC group and neuropsychology.

## Discussion

In the present study, we have observed a pattern of cortical thinning associated with progressive cognitive deterioration that involves the medial temporal lobes and posterior medial cortical regions. Areas with decreased cortical thickness are present in PD-MCI patients, and correspond to central nodes of large-scale cortical networks functionally specialized in declarative memory (entorhinal cortex, anterior temporal pole), semantic knowledge (parahippocampus, fusiform gyrus), and visuoperceptive integration (bSTS, lingual gyrus, cuneus and precuneus) [34], [35], [36]. Based on our findings, decrease in cortical thickness in PD seems to relate to concurrent atrophy of areas playing an important role in the storage of prior experiences, integration of external perceptions, and semantic processing [34], [37].

To our knowledge, this is the first study to analyze changes in cortical thickness in PD patients with normal cognition, mild cognitive impairment and dementia. The changes observed in our sample are very similar to those described in three recently published papers that utilized MRI and voxel-based morphometry searching for changes in grey matter volume across these stages of cognitive decline [16], [17], [38]. In the Weintraub et al. study [16], decreased volumes in the hippocampus and parietal–temporal cortex were observed in PD-MCI patients, and is more evident in the PDD group. Melzer et al. [17] showed also a linear progression in grey matter volume loss across cognitive stages, with initial atrophy of the temporal, parietal, frontal and caudal hippocampus in PD-MCI, that became more extensive in PDD patients, and additional volume decreases in the parahippocampus, lingual gyrus, and posterior cingulate gyrus. Interestingly, cortical changes correlated with the degree of global cognitive dysfunction, but did not correlate with motor impairment. Our findings of a significant relationship between delayed verbal memory and cortical thinning in the bilateral anteromedial temporal cortex support previous studies in which memory impairments in PD related to hippocampal volume loss [11], [16].

In these studies, however, no cortical differences were found between HC and PD with normal cognition. Song et al. found loss of gray matter in PD-NC patients in the left occipital area, extending to the bilateral temporal, left prefrontal, and right occipital areas in PD-MCI patients [15]. Yet, the authors did not report correlations of cortical changes with neuropsychology.

In our sample, we observed very early cortical thinning in the PD-NC group in the middle temporal gyrus bilaterally, left superior frontal gyrus, right isthmus (posterior) cingulate cortex, right inferior parietal lobule, and lateral occipital cortex. These findings may be explained by the greater sensitivity of imaging/postprocessing techniques [19], [20], and seem to indicate that some structural changes can be detected by cortical thickness well before the cortical atrophy associated with cognitive impairment. In fact, these changes did not correlate with any neuropsychological task, and PD-NC patients did not differ significantly from the HC group in any cognitive item. In the PD-MCI and PDD groups, correlations found between neuropsychology and decreased cortical thickness indicate the importance of cortical thinning of temporal and parietal regions in the performance of attentional, mnesic and visuoperceptive tasks. In particular, sustained attention and alternating verbal fluency, both of which have been consistently reported as two of the earlier tasks to become impaired in PD [7], [39], correlated with thinning in the cuneus, bSTS, fusiform gyrus, and lingual gyrus in both PD-MCI and PDD patients. Immediate free-recall memory, that relies more on attentional resources than in storage capacities, also correlated to posterior thinnig (cuneus) in PD-MCI, while in PDD delayed free-recall memory correlated with thinning in the right anteromedial temporal cortex, right parahippocampus, and left lingual gyrus. Regarding the two posterior cortical tasks of the PD-CRS, confrontation naming correlated with thinning in the fusiform gyrus in PD-MCI, and with thinning in the fusiform gyrus, temporal pole, and parahippocampus in PDD patients. Clock copying correlated with decreased cortical thickness in the precuneus, parahippocampus, and lingual gyrus in PDD.

The discrete changes that we have found in neocortical regions may suggest that structural changes in PD play only an additive dysfunctional effect on cortical areas already impaired by metabolic defects and presynaptic dysfunction [40]. However, the relatively limited atrophic changes observed are centered in regions representing important nodes of information integration [37], [41], therefore apparently minor structural changes may entail important functional impairments in distant but functionally related areas. The medial temporal lobe (including the entorhinal cortex and anteriomedial temporal cortex) provides information from prior experiences in the form of memories and associations between stimuli from different sensory modalities [42]. In both Alzheimer’s disease (AD) and dementia with Lewy bodies, concurrent atrophy of the medial temporal lobe, precuneus, and temporo-parietal cortices appears to play a major role in the development of dementia [43]. Cortical thickness analysis of normal brains has revealed the existence of a set of posterior medial cortical regions that form a densely interconnected network [44]. Key components of this network are the cuneus, precuneus, posterior aspects (isthmus) of the cingulate cortex, inferior parietal lobule, and the bSTS. These heteromodal cortical areas have a central role in integrating information across functionally segregated brain regions [45], and seem particularly vulnerable to atrophy in different neurodegenerative diseases [43], [45]. Noteworthy, these posterior cortical regions are densely interconnected with the medial temporal lobe [46]. In our sample, the areas with the more robust projections to the entorhinal cortex, namely, the parahippocampus, lingual gyrus, and bSTS, were precisely those to become progressively more atrophic in the transition from normal cognition to dementia. These regions respond to multiple sensory stimuli and provide multimodal sensory information to the hippocampal formation [47], [48].

There is no clear data on the potential underlying mechanisms of cortical thinning as detected by Freesurfer on MRI. However, literature based on histology suggests that cortical thinning is unlikely to originate predominantly from neuronal death. Rather, cellular shrinkage and reduction in dendritic arborization are more likely to account for cortical thinning. [49].

The increased cortical thickness observed in the caudal (PD-MCI vs PD-NC) and rostral (PDD vs. PD-MCI) regions of the anterior cingulate cortex (ACC), the correlation between thicker caudal ACC and impaired sustained attention, and the posterior thinning of the caudal ACC in the PDD group, are in line with previous findings of progressive thickness changes in neurodegenerative diseases analyzed over time. For instance, in the preclinical and early phases of AD the evolution of cortical thickness appears to present an inverted-U shape model, with initial increased cortical thickness in the ACC and temporoparietal areas, and posterior thinning of these same structures. [50] In healthy ApoE-4 carriers, increased thickness has been associated with impaired selective attention [51] and, interestingly, neuropathological studies of healthy elderly subjects compared to asymptomatic AD subjects and patients with AD-MCI have shown the existence of a phase of hypertrophy of the neuronal cell bodies in preclinical AD that precedes the atrophy of these areas in symptomatic patients. [52] Similarly, patients with early Huntington’s disease present specific increased thickness in the ACC that progresses to neuronal atrophy in more advanced stages. [53].

We acknowledge some limitations of our study. First, lack of significance at the corrected level relies mostly on a limited sample size. All p-values reported in relation to cortical thinning in this sample are uncorrected, and therefore the results of this study need to be replicated in future studies. Second, there are some technical limitations in cortical thickness determinations on MRI due to artefacts, such as susceptibility or motion, which can make the brain surfaces difficult to be defined. In this regard, the method that we have implemented with Freesurfer, which uses an automatic approach, and a manual correction afterwards, seems appropiate to minimize this limitation. Thirdly, we used a hypothesis-driven approach to search for correlations between cortical thinning and neuropsychological performance. This approach is based on the changes observed between cognitive groups, looking for those cognitive domains that characterize better patients with PD-MCI or PDD. Since causality cannot be inferred by correlational findings alone, and larger samples are needed to look for correlations that survive multiple comparisons correction, future studies are warranted to verify our results. Finally, the presence of hallucinations in the PDD group may confound the results of the study. Imaging studies have associated the presence of visual hallucinations with grey matter volume reductions in the orbitofrontal cortex, parahippocampal area, lingual gyrus, and superior parietal lobe. [54], [55] Studies analyzing PDD patients with and without hallucinations are then needed to disentangle the differential involvement of these areas on cognitive deterioration and hallucinatory phenomena.

In summary, our work suggests that concurrent thinning of areas involved in memory systems, semantic networks, and visuoperception may explain the important clinical impact that restricted areas of atrophy may have on the processing of stimuli coming from different sensory modalities, which is essential for the elaboration of high-order sensory representations of the external and internal world [41].
